# Do differences in emergency medical services (EMS) response time to an arrest account for the survival differences between EMS-witnessed and bystander-witnessed out of hospital cardiac arrest?

**DOI:** 10.1016/j.resplu.2024.100696

**Published:** 2024-06-25

**Authors:** David Majewski, Stephen Ball, Milena Talikowska, Jason Belcher, Rudolph Brits, Judith Finn

**Affiliations:** a Prehospital, Resuscitation and Emergency Care Research Unit (PRECRU), Curtin School of Nursing, Curtin University, Bentley, Western Australia, Australia; b St John WA, Belmont, Western Australia, Australia; c Medical School (Emergency Medicine), The University of Western Australia, Nedlands, Western Australia, Australia; d School of Public Health and Preventive Medicine, Monash University, Melbourne, Victoria, Australia

**Keywords:** Out-of-hospital cardiac arrest, EMS-witnessed, Bystander-witnessed, Survival

## Abstract

**Introduction:**

Out-of-hospital cardiac arrests (OHCA) witnessed by Emergency Medical Services (EMS) are reported to have more favourable survival than bystander-witnessed arrests, even after adjusting for patient and arrest factors known to be associated with increased OHCA survival. This study aims to determine whether the survival advantage in EMS-witnessed arrests can be attributed to differences in the EMS response time to the arrest.

**Methods:**

Using registry data we conducted a retrospective, population-based cohort study of bystander- and EMS-witnessed OHCAs of medical aetiology who received an EMS resuscitation attempt in Western Australia between 2018–2021. EMS response time to arrest was assumed to be zero for EMS-witnessed arrests. Multivariable logistic regression was used to compare 30-day OHCA survival by witness and bystander CPR (B-CPR) status, adjusting for EMS response time to arrest, and patient and arrest characteristics.

**Results:**

Of 2,130 OHCA cases, 510 (23.9%) were EMS-witnessed and 1620 were bystander-witnessed: 1318/1620 (81.4%) with B-CPR, and 302/1620 (18.6%) with no B-CPR. The median EMS response time to bystander-witnessed arrests who received B-CPR was 9.9 [Q1,Q3: 7.4, 13.3] minutes. After adjusting for the EMS response time and patient and arrest factors, 30-day survival remained significantly lower in both the bystander-witnessed group with B-CPR (aOR 0.56; 95% CI 0.34 – 0.91) and bystander-witnessed group without B-CPR (aOR 0.23; 95% CI 0.11 – 0.46).

**Conclusion:**

An increased EMS response time does not fully account for the higher OHCA survival in EMS-witnessed arrests compared to bystander-witnessed arrests.

## Introduction

Out-of-hospital cardiac arrests (OHCA) that first occur in the presence of emergency medical services (EMS) personnel (i.e. EMS-witnessed OHCA) make up approximately 11% of all OHCA,^1^ with this proportion reported to be increasing over time.^2^, ^3^ Compared to bystander-witnessed arrests, EMS-witnessed arrests are more likely to present with patient and arrest characteristics that are less favourable for OHCA survival; such as increased patient age, higher proportion of arrests in non-public locations, and increased rates of non-shockable arrest rhythms.^4^, ^5^ Despite this, patients with EMS-witnessed arrests are reported to have higher rates of survival than those with bystander-witnessed arrests.^4^, ^5^, ^6^ This survival advantage has been proposed to be a result of immediate EMS resuscitative care and, by extension, earlier advanced hospital care.^6^ Using the Western Australian OHCA registry, this study aims to determine whether differences in 30-day survival between EMS-witnessed and bystander-witnessed OHCA can be fully explained by the delays in EMS-attendance to the arrest patient, after adjusting for patient and arrest factors known to be associated with survival.^7^

## Methods

### Study design

We conducted a population-based, retrospective cohort study of St John WA (SJ-WA) EMS-attended OHCA in Western Australia (WA) between 1st January 2018 and 31st December 2021. Patients were included in the study if: their arrest was either EMS- or bystander-witnessed; they were 18 years of age or older at the time of arrest; their arrest was of presumed medical aetiology^8^; and they received an attempted resuscitation from EMS personnel. Patients who arrested during interhospital transfers were excluded from the study as these patients are sometimes under the clinical management of hospital medical staff (i.e. nurse or doctor) during the transport*.* The primary outcome of interest was 30-day survival. The primary exposure of interest was EMS response time to the arrested patient. For EMS-witnessed arrests, the EMS response time to the arrested patient was assumed to be zero (as they arrested in the presence of EMS personnel), while for bystander-witnessed arrests it was approximated using the EMS scene response time.

### Study setting

Western Australia is the largest state by area in Australia, covering 2.53 million square kilometres.^9^ In 2022, WA had a population of 2.8 million people^10^ with 79% residing in the greater Perth metropolitan area.^11^ SJ-WA is the sole provider of road EMS services for the state, and during the study period operated a single-tiered Advanced Life Support (ALS) ambulance in metropolitan Perth (staffed by paramedics) and a mix of ALS and Basic Life Support (BLS) ambulances in rural and remote areas (staffed by paramedics, volunteer ambulance officers, or a mix).^12^ The SJ-WA ambulance call centre utilises the Medical Priority Dispatch System (MPDS). Except for cases that are dispatched as obvious or expected deaths (which are very unlikely in our study due to the cohort being restricted the cases with an EMS resuscitation attempt),^13^ the MPDS directs the emergency call-taker to provide telephone guided CPR for all cases of cardiac arrest, even in the instance that CPR is already being provided by bystanders. Unless resuscitative efforts are ceased in the field due to futility or a not-for-resuscitation (NFR) order,^14^ OHCA patients are transported to the nearest appropriate hospital.

### Data sources

Data was sourced from the SJ-WA OHCA Registry,^15^ which contains details for all metropolitan OHCAs attended by SJ-WA EMS since 1996 and all rural OHCAs since 2014. The registry is maintained by the Prehospital, Resuscitation and Emergency Care Research Unit (PRECRU) at Curtin University; extracting data from the electronic patient care record (e-PCR) completed by SJ-WA EMS personnel and linked to computer-aided dispatch data. The OHCA registry contains information on patient demographics, arrest characteristics, EMS response and transport time intervals, pre-ambulance care by bystanders, and EMS interventions. The ‘Utstein’ prognostic variables^8^ in the registry include patient age and sex, OHCA witnessed status (bystander-witnessed; EMS-witnessed; unwitnessed), bystander cardiopulmonary resuscitation (B-CPR), EMS response time (call answer to arrival on scene), initial cardiac arrest rhythm (shockable/non-shockable determined by AED use, else as recorded by paramedics from the defibrillator monitor), return of spontaneous circulation (ROSC) in the field, and ROSC on arrival at the Emergency Department (ED). Arrest aetiology is determined by PRECRU research staff and is based on a combination of the free text section of the ePCR describing the event and EMS dispatch problem codes. Thirty-day survival following OHCA was determined by manual look-up in the WA Death Registry, for those patients not recorded as deceased by SJ-WA EMS personnel.

### Statistical analysis

Since the provision of bystander CPR (B-CPR) has been shown to double the likelihood of survival in OHCA,^16^ we stratified the bystander-witnessed group into those who did and did-not receive B-CPR, as done by previous studies.^4^, ^6^ The study population was therefore stratified into three groups based on arrest witness status and provision of bystander CPR as follows: EMS-witnessed, bystander-witnessed with B-CPR and bystander-witnessed without B-CPR. In addition to EMS response time to the arrested patient (in minutes), other patient and arrest characteristics were summarised (by the three groups) using counts and percentages for categorical data and medians (with first and third quartiles i.e. Q1, Q3) reported for continuous data. Patient and arrest characteristics examined in this study included patient age (years), sex, arrest location (public, non-public), initial shockable arrest rhythm (yes, no), dispatch priority (1–3 with ‘1’ being lights and sirens), EMS dispatch problem (‘chief complaint’ based on the Medical Priority Dispatch System, MPDS Version 13),^17^ EMS on scene time (in minutes), EMS scene-to-ED time (in minutes), region (metropolitan Perth, rural), any attainment of ROSC, ROSC on arrival to ED, and 30-day survival. ‘EMS scene time’ represents EMS on-scene time while ‘EMS scene-to-ED time’ represents the time between EMS scene departure and EMS arrival at ED. Regarding arrest location (as public or non-public), EMS-witnessed arrests that first occurred within the ambulance were coded based on the location that emergency services were originally dispatched to. Initial arrest rhythm (ventricular fibrillation [VF], ventricular tachycardia [VT], pulseless electrical activity [PEA] and asystole) was provided for cases where such detail was available. Finally, we compared the use of advanced airways (endotracheal tube, laryngeal mask airway or i-Gel) and administration of resuscitation drugs (adrenaline, amiodarone) between the groups.

For statistical testing, we were specifically interested in two comparisons: i) EMS-witnessed vs bystander-witnessed with B-CPR and, ii) EMS-witnessed vs bystander-witnessed without B-CPR. Our null hypothesis was that there was no difference in specified patient and arrest characteristics between EMS-witnessed arrests and bystander-witnessed arrests (with or without B-CPR). Statistically significant differences were identified using either the Chi-squared/Fisher exact test for categorical data or the *t* test/ Mann-Whitney *U* test for continuous data. A Bonferroni Correction^18^ (α = 0.025) was used to correct for there being two comparisons for each variable (EMS-witnessed versus bystander-witnessed with CPR; EMS-witnessed versus bystander-witnessed without CPR).

Crude associations between witness status group (EMS-witnessed, bystander-witnessed with B-CPR, bystander-witnessed without B-CPR) and 30-day OHCA were examined using logistic regression. Multivariable logistic regression was used to examine the association between witness status group (EMS-witnessed, bystander-witnessed with B-CPR, bystander-witnessed without B-CPR) and 30-day OHCA survival, while adjusting for i) patient and arrest factors and, ii) patient and arrest factors and time to EMS attendance of the arrested patient (zero minutes for EMS-witnessed arrests). Patient and arrest characteristics included in the model (using the ‘Enter’ method,^19^ i.e. simultaneous inclusion of variables) were: patient age (in years), patient sex (female/male), region (rural/metropolitan Perth), initial monitored rhythm (non-shockable/shockable), arrest location (public/non-public) and EMS response time to arrested patient (in minutes). Data analyses were performed using SPSS v29 (IBM Inc., Armonk, NY, USA). Results for analyses were considered statistically significant if p < 0.05 or p < 0.025 where a Bonferroni Correction for multiple group testing was applied.

### Ethics

This study was approved by the Human Research Ethics Committee (HREC) of Curtin University as a sub-study of the over-arching project HR128/2013; and approved by the St John WA Research Governance Committee.

## Results

**Fig. 1:**
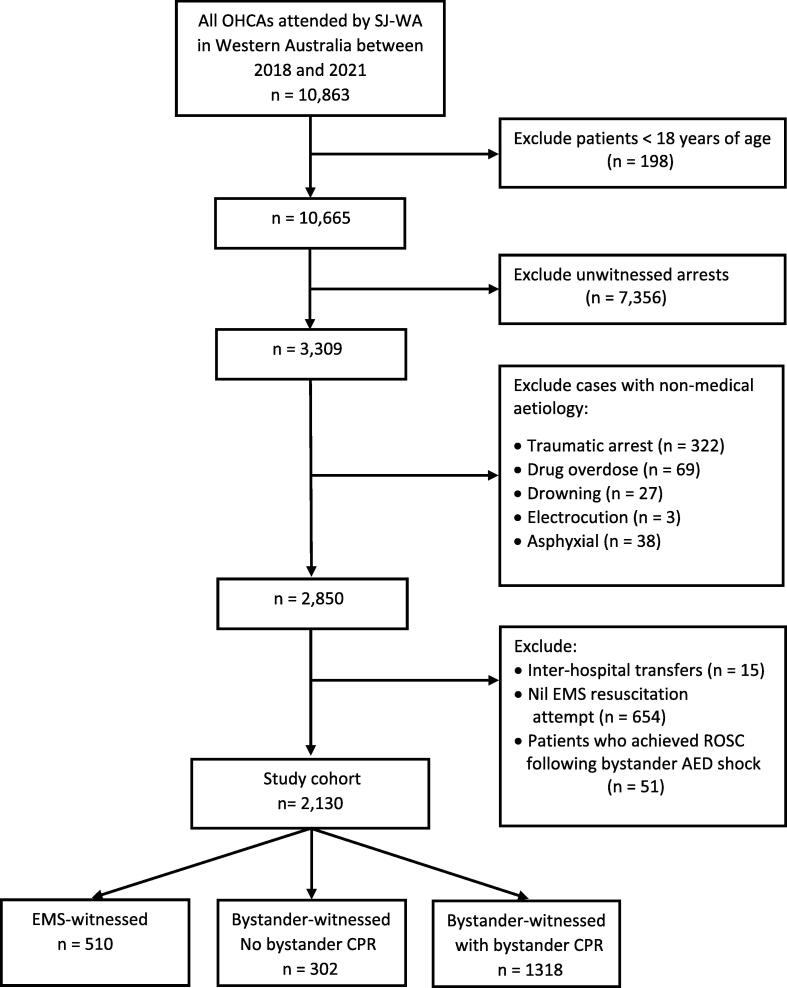
Flow diagram of included study cohort.

There were 10,863 cases of OHCA attended by EMS in Western Australia between 2018 and 2021. After excluding cases where patients were < 18 years of age, had an unwitnessed arrest, had an arrest of non-medical aetiology, arrested during interhospital transfer, achieved ROSC following bystander AED shock or where no resuscitation was attempted by EMS, a total of 2,130 cases remained and were included in our final study cohort (Fig. 1). This consisted of 510 (23.9%) patients who had an EMS-witnessed arrest and 1620 who had a bystander-witnessed arrest: 1318/1620 (81.4%) with B-CPR, and 302/1620 (18.6%) with no B-CPR.

### Patient and arrest characteristics

Table 1 shows the baseline characteristics of the study cohort, stratified by the three arrest witness status and B-CPR provision groups. The median EMS response time to arrested patient was 9.9 [Q1,Q3: 7.4, 13.3] minutes for bystander-witnessed arrests with B-CPR and 9.2 [Q1,Q3: 7.2, 12.0] minutes for bystander-witnessed arrests without B-CPR. There were significant differences between the EMS-witnessed group and bystander-witnessed with B-CPR group across all patient/arrest characteristics. Compared to patients who had an EMS-witnessed arrest, those with a bystander-witnessed arrest with B-CPR tended to be younger (median age: 66 vs 70 years; p < 0.001), more likely to be: male (74.4% vs 63.7%; p < 0.001), present with a shockable rhythm (47.5% vs 31.9%; p < 0.001), and arrest in a public location (26.4% vs 9.6%; p < 0.001). Of the cases where the actual initial arrest rhythm (i.e. VF, VT, PEA, asystole) could be identified (i.e. 469/510 [92%] EMS-witnessed and 1108/1318 [84%] bystander witnessed with B-CPR), bystander-witnessed arrests with B-CPR presented with a lower proportion of PEA as the initial arrest rhythm (15.6% vs 44.8%; p < 0.001) and a higher proportion of asystole (40.6% vs 23.0%; p < 0.001) compared to the EMS-witnessed arrests. A post hoc sensitivity analysis was conducted to explore the effect of unknown initial rhythm data (i.e. VF/VT/PEA/Asystole) on this relationship (supplementary table 2). The sensitivity analysis showed that if all non-shockable cases with a missing initial rhythm maximized PEA in bystander-witnessed arrests and minimized PEA in EMS-witnessed cases (and vice-versa with respect to asystole), the proportion of PEA rhythms would remain significantly lower in the bystander-witnessed group than the EMS-witnessed group (17.5% vs 42.2%; p < 0.005)*.* Differences in interventions between EMS-witnessed and bystander-witnessed groups is shown in Supplementary table 1. Compared to EMS-witnessed arrests, bystander-witnessed arrests with B-CPR were significantly more likely to receive an advanced airway (71.9% vs 50.8%; p < 0.001) and/or resuscitation drugs during the arrest (71.9% vs 50.0%; p < 0.001). There were also statistically significant differences across most patient and arrest characteristics between the bystander-witnessed without B-CPR group and the EMS-witnessed group, although the magnitude of these differences tended to be smaller.

**Table 1:** The patient and arrest characteristics of OHCAs in Western Australia, stratified by arrest witness status.

		**Arrest witness status**				
		**EMS-witnessed** **(n = 510)**	**Bystander-witnessed** **No bystander CPR** **(n = 302)**	**p-value**^a^	**Bystander-witnessed** **with bystander CPR** **(n = 1318)**	**p-value**^b^
Patient Age	Median [Q1, Q3)	70 [58, 79]	70 [57, 80]	P = 0.661	66 [55, 76]	P < 0.001*
Sex	Male, n (%)	325 (63.7%)	207 (68.5%)	P = 0.17	981 (74.4%)	P < 0.001*
Arrest location	Public	49 (9.6%)	27 (8.9%)	P = 0.804	348 (26.4%)	P < 0.001*
	Non-public	461 (90.4%)	275 (91.1%)		970 (73.6%)	
Presumed cardiac aetiology		481 (94.3%)	289 (95.7%)	P < 0.418	1288 (97.7%)	P < 0.001*
EMS response time to arrested patient (minutes)	Median [Q1, Q3)	0.0 [0.0,0.0]	9.3 [7.2, 12.1]	P < 0.001*	9.9 [7.4, 13.3]	P < 0.001*
EMS scene time (minutes)	Median [Q1, Q3)	33.6 [24.3,47.5]	41.1 [31.4,109.8]	P < 0.001*	33.9 [25.3,72.5]	0.156
EMS scene-to-ED time (minutes)	Median [Q1, Q3)	10.9 [7.0,16.2]	8.4 [5.2,11.7]	P < 0.001*	9.6 [6.1,14.0]	P < 0.001*
Shockable initial arrest rhythm	Yes	159 (31.9%)	71 (23.9%)	P = 0.019*	620 (47.5%)	P < 0.001*
	No	339 (68.1%)	226 (76.1%)		684 (52.5%)	
Initial Arrest Rhythm^c^	VF	131 (27.9%)	62 (22.4%)	P < 0.001*	474 (42.8%)	P < 0.001*
	VT	20 (4.3%)	3 (1.1%)		7 (0.6%)	
	PEA	210 (44.8%)	81 (29.2%)		174 (15.6%)	
	Asystole	108 (23.0%)	131 (47.3%)		453(40.9%)	
Dispatch priority	1	460 (90.2%)	289 (95.7%)	P = 0.018	1315 (99.8%)	P < 0.001*
	2	40 (7.8%)	10 (3.3%)		1 (0.1%)	
	3	10 (2.0%)	3 (1.0%)		2 (0.2%)	
Geographic location	Metro	433 (84.9%)	241 (79.8%)	P = 0.067	997 (75.6%)	P < 0.001*
	Rural	77 (15.1%)	61 (20.2%)		321 (24.4%)	
ROSC any	Yes	245 (48.0%)	79 (26.2%)	P < 0.001*	475 (36.0%)	P < 0.001*
ROSC at hospital	Yes	199 (39.0%)	52 (17.2%)	P < 0.001*	370 (28.1%)	P = 0.001*

Abbreviations: AED = automated external defibrillator; CPR = cardiopulmonary resuscitation; ED = emergency department; EMS = emergency medical services; PEA = pulseless electrical activity; ROSC = return of spontaneous circulation; VF = ventricular fibrillation; VT = ventricular tachycardia.

a P-value testing for difference between EMS-witnessed arrests and bystander-witnessed arrests with no bystander CPR.

b P-value testing for difference between EMS-witnessed arrests and bystander-witnessed arrests with bystander CPR.

c Specific initial rhythm (VF, VT, PEA or Asystole) unknown for some cases where AED was used: 41 in EMS-witnessed group; 25 unknown in bystander witnessed (without B-CPR) group; 210 cases in bystander witnessed (with B-CPR) group.

* Statistically significant result after Bonferroni Correction using.$\propto_{\mathrm{adjusted}}=0.025$

### Thirty-day survival

Overall, 30-day OHCA survival was 27.3% in the EMS-witnessed group, 17.5% in the bystander-witnessed group with B-CPR and 5.0% in the bystander-witnessed group without B-CPR. The results of the unadjusted and adjusted logistic regression model examining 30-day survival are presented in Table 2. Compared to the EMS-witnessed group, crude 30-day survival was significantly lower in both the bystander-witnessed group with B-CPR (OR 0.57, 95% CI: 0.45 – 0.72), and the bystander-witnessed group without B-CPR (OR 0.14, 95% CI: 0.08 – 0.24). After adjusting for patient and arrest factors only, 30-day survival was lower in both the bystander-witnessed group with B-CPR (aOR 0.23; 95% CI 0.17 – 0.32) and the bystander-witnessed group without B-CPR (aOR 0.11; 95% CI 0.06 – 0.20). After adjusting for patient and arrest factors and EMS response time to the arrested patient, 30-day survival remained lower in both the bystander-witnessed group with B-CPR (aOR 0.56; 95% CI 0.34 – 0.91) and the bystander-witnessed group without B-CPR (aOR 0.23; 95% CI 0.11 – 0.46).

**Table 2:** Crude and Adjusted Odds Ratios (95% CI) for 30-day OHCA survival.

		**Crude** **OR (95% CI)**	**Adjusted Model 1**^a^ **aOR (95% CI)**	**Adjusted Model 2**^b^ **aOR (95% CI)**
**Arrest witness status**				
	EMS-witnessed	ref	ref	ref
	Bystander-witnessed + bystander CPR	0.57 (0.45 – 0.72)	0.23 (0.17 – 0.32)	0.56 (0.34 – 0.91)
	Bystander-witnessed − bystander CPR	0.14 (0.08 – 0.24)	0.11 (0.06 – 0.20)	0.23 (0.11 – 0.46)
**Patient Age**	(per year)	0.98 (0.97 – 0.98)	0.98 (0.97 – 0.99)	0.98 (0.97 – 0.99)
**Patient sex**	Male	1.49 (1.15 – 1.94)	0.99 (0.73 – 1.36)	1.00 (0.73 – 1.37)
**Regional location of arrest**				
	Metropolitan	ref	ref	ref
	Rural/regional	0.50 (0.37 – 0.67)	0.47 (0.33 – 0.68)	0.64 (0.44 – 0.93)
**Arrest location**				
	Public	ref	ref	ref
	Non-public	0.34 (0.27 – 0.44)	0.43 (0.32 – 0.58)	0.45 (0.33 – 0.61)
**Initial rhythm**				
	VF/VT	ref	ref	ref
	PEA/Asystole	0.08 (0.06 – 0.10)	0.08 (0.06 – 0.11)	0.08 (0.06 – 0.11)
**EMS response time to arrested patient**	(per minute)	0.94 (0.92 – 0.95)		0.92 (0.88 – 0.95)

Abbreviations: aOR = adjusted odds ratio; CPR = cardiopulmonary resuscitation; EMS = emergency medical services; OR = odds ratio; PEA = pulseless electrical activity; ref = reference category; VF = ventricular fibrillation; VT = ventricular tachycardia.

a Model 1 adjusted for: patient age, sex, Regional location of arrest, arrest location and initial rhythm.

b Model 2 adjusted for: patient age, sex, Regional location of arrest, arrest location, initial rhythm and EMS response time to arrest.

## Discussion

In our study cohort, 30-day OHCA survival was statistically significantly higher for EMS-witnessed arrests (27.3%), compared to bystander-witnessed arrests (17.5%) with B-CPR and bystander-witnessed arrests without B-CPR (5.0%). After adjusting for EMS delay in attending bystander-witnessed arrests (as well as adjusting for patient and arrest characteristics), bystander-witnessed arrests continued to have less favourable survival odds compared to EMS-witnessed arrests. To our knowledge, our study is the first that attempts to adjust for the delay in EMS attendance for bystander-witnessed arrests, when making comparisons with EMS-witnessed arrests.

There were significant differences in patient and arrest characteristics between the bystander-witnessed with B-CPR group and the EMS-witnessed group, although these differences − such as younger patient age, higher proportion of arrests in a public location and higher proportion of arrests with shockable rhythms − would have been expected to favour 30-day survival in the bystander-witnessed group.^20^, ^21^ Indeed, after adjusting for patient and arrest characteristics we found the survival odds for bystander-witnessed arrests (relative to EMS-witnessed arrests) were further reduced from our unadjusted model (OR 0.23 vs OR 0.57 respectively). Additionally, we found that bystander-witnessed arrests were more likely than EMS-witnessed arrests to receive either advanced airway management and/or cardiac arrest drugs during the resuscitation. We hypothesise that EMS-witnessed arrests may achieve ROSC sooner than bystander-witnessed arrests, thereby providing less opportunity for interventions to be performed. This has been described as ‘resuscitation time bias’,^22^ where an intervention is more likely to occur the longer the cardiac arrest continues; and the length of resuscitation is strongly associated with worse outcome.^22^

Our results, are consistent with those reported by others.^4^, ^5^, ^23^ A 2015 study from Victoria, Australia^4^ reported survival to hospital discharge as 34.9% for EMS-witnessed arrests and 21.1% for bystander-witnessed arrests with B-CPR. This study also reported that patients with bystander-witnessed arrests who received B-CPR tended to be younger (median age 67 vs 72 years), more often male (73.3% vs 66.4%) and more likely to present with a shockable rhythm (59.7% vs 41.5%) than patients who had EMS-witnessed arrests. After adjustment for potential confounders, the authors reported that patients with bystander-witnessed arrests with B-CPR had lower odds of 30-day survival (OR 0.16; 95% CI, 0.13 – 0.19) than EMS-witnessed arrests.^4^ This was comparable, albeit lower, than the OR reported in our current study (OR 0.23; 95% CI, 0.17 – 0.32). A 2010 study^6^ from the United States similarly reported higher survival (to hospital discharge) in EMS-witnessed arrests (18%) than bystander-witnessed arrests with B-CPR (15%) or bystander-witnessed arrests without B-CPR (10%). This study also reported that bystander-witnessed arrests were more likely to occur in public locations or present with shockable arrest rhythms compared to EMS-witnessed arrests. After adjustment for potential confounders, the authors reported that patients with bystander-witnessed arrests with B-CPR had lower odds of survival to hospital discharge (OR 0.41; 95% CI 0.36 – 0.43) than EMS-witnessed arrests.

In our study, the proportion of non-shockable arrest rhythms was significantly greater in the EMS-witnessed patient group than the bystander-witnessed with B-CPR group, despite the absence of any delay to rhythm monitoring in the EMS-witnessed group. The reason for this variation is unclear, although one possibility is that the difference in initial rhythm is due to the underlying pathology of the arrest. The Ontario Prehospital Advanced Life Support (OPALS) study^24^ reported that on average, patients who had EMS-witnessed arrests and presented with non-shockable rhythms, were more likely to experience pre-arrest dyspnoea and less likely to experience pre-arrest chest pain. A Victorian study^25^ which reported on the association between pre-arrest symptoms and initial arrest rhythm in EMS-witnessed OHCA found patients with pre-arrest chest pain had increased odds of an initial shockable rhythm while those with pre-arrest dyspnoea had increased odds of a non-shockable rhythm. A post hoc analysis of primary dispatch complaint in our own EMS-witnessed patient group showed a similar pattern; EMS-witnessed arrests with a primary dispatch complaint of chest pain were more likely to present in a shockable rhythm and those with a primary dispatch complaint of dyspnoea were more likely to present in a non-shockable rhythm. It is also possible that the variation in initial rhythm between EMS- and bystander-witnessed arrests is not due to differences in patient case mix, but rather a result of delays in first monitoring the initial rhythm. Unlike EMS-witnessed arrests, where the primary arrest rhythm is recorded, delays in monitoring the rhythm of bystander-witnessed arrests could potentially mean that the recorded rhythm represents some deviation away from the primary arrest rhythm.^26^, ^27^ This could, as suggested by Holmström et al, account for the higher proportion of asystole seen in bystander-witnessed arrests.^28^ Finally, although the proportion of non-shockable arrest rhythms was greater in the EMS-witnessed group than the bystander-witnessed group, the EMS-witnessed group had a greater proportion of PEA and a lower proportion of asystole than the bystander-witnessed (with B-CPR) group. As PEA rhythms tend to have more favourable survival outcome than asystolic arrests,^29^ it is possible that the higher survival in the EMS-witnessed group could, in part, be due to the greater proportion of PEA and lower proportion of asystole.

Although our study found that the delay in EMS response time to bystander-witnessed OHCA patients compared to EMS-witnessed arrests was associated with reduced 30-day survival, adjusting for this delay, as well as patient and arrest characteristics, failed to fully explain the difference in survival between EMS-witnessed and bystander-witnessed OHCA. Given the negative association between pre-arrest comorbidity and OHCA survival,^30^ we suspect that differences in patient pre-arrest comorbidity may account for some of the remaining survival discrepancy. Studies comparing pre-arrest comorbidity among EMS-witnessed and bystander-witnessed arrests are rare, although a 2022 review^28^ suggests that pre-arrest cardiovascular and/or respiratory disease may be more common amongst EMS-witnessed arrests. Previous studies exploring OHCA survival and arrest witness status have not adjusted for differences in patient pre-arrest comorbidity. We suspect this is due to difficulties in EMS obtaining comprehensive comorbidity data for OHCA patients.

### Limitations

Our study had several limitations. Firstly, we approximated ‘EMS response time to the arrested patient’ using ‘EMS scene response time’ for bystander-witnessed arrests, while EMS-witnessed arrests were assigned a response time of zero (as EMS were present at the time of arrest). It is likely our approximation underestimated the time in cases of bystander-witnessed arrest, as we did not account for the additional time required to physically reach the patient’s side from the ambulance vehicle (EMS response time is logged automatically when the ambulance reaches the dispatched GPS coordinates). Additionally, the ‘time to EMS attendance of arrested patient’ variable assumes bystander-witnessed patients arrested close to the time the emergency call to EMS was placed by the bystander. However, there may have been unmeasured delays between the arrest and placement of the emergency call, and some patients may have arrested after the call was placed but prior to EMS arrival.

Secondly, as some OHCA cases (in rural areas) were attended by EMS with AEDs (instead of manual defibrillators), we classified patient initial arrest rhythm as either shockable or non-shockable for the multivariable regression models to have a consistent classification across our cohort. Thirdly, our study did not report patient pre-arrest comorbidity. Patient pre-arrest comorbidity recorded by ambulance personal is generally considered unreliable^30^; particularly in cases of bystander-witnessed arrest where comorbidity data can only be elicited from a bystander who may have little to no knowledge of a patient’s medical history. Lastly, EMS scene and EMS scene-to-ED times for EMS-witnessed arrests may have been influenced by both the patient’s condition prior to arrest, and the time to arrest (after EMS arrival on scene). As such, EMS scene and EMS scene-to-ED times were not included in the multivariable regression models.

## Conclusion

EMS-witnessed arrests have more favourable survival outcomes than bystander-witnessed arrests (irrespective of B-CPR status); even after adjusting for known patient and arrest factors and the effect of EMS response time to bystander-witnessed arrests. Our finding suggests that ‘other’ unmeasured factors (e.g. patient comorbidity, arrest aetiology, etc) may account for the differences in survival between EMS-witnessed and bystander-witnessed OHCA.

## Funding

Judith Finn is the recipient of a National Health and Medical Research Council (NHMRC) Investigator Grant #1174838. The Prehospital, Resuscitation and Emergency Care Research Unit (PRECRU) receives research funding from St John Western Australia (SJWA).

## CRediT authorship contribution statement

**David Majewski:** Writing – review & editing, Writing – original draft, Validation, Methodology, Formal analysis, Data curation, Conceptualization. **Stephen Ball:** Writing – review & editing, Writing – original draft, Validation, Methodology, Formal analysis, Data curation, Conceptualization. **Milena Talikowska:** Writing – review & editing, Data curation. **Jason Belcher:** Writing – review & editing, Validation, Resources, Methodology, Conceptualization. **Rudolph Brits:** Writing – review & editing, Conceptualization. **Judith Finn:** Writing – review & editing, Validation, Supervision, Methodology, Funding acquisition, Formal analysis, Conceptualization.

## Declaration of competing interest

The authors declare the following financial interests/personal relationships which may be considered as potential competing interests: Jason Belcher and Rudolph Brits are employees of SJWA. Judith Finn and Stephen Ball hold adjunct research positions with SJWA. David Majewski, Milena Talikowska, Judith Finn and Stephen Ball are employees of the Prehospital, Resuscitation and Emergency Care Research Unit (PRECRU) at Curtin University; PRECRU receives research funding from SJWA. There are no other conflicts of interest to declare.

## References

[1] Gowens P., Smith K., Clegg G., Williams B., Nehme Z. (2022). Global variation in the incidence and outcome of emergency medical services witnessed out-of-hospital cardiac arrest: A systematic review and meta-analysis. Resuscitation.

[2] Majewski D., Ball S., Bailey P., Bray J., Finn J. (2022). Trends in out-of-hospital cardiac arrest incidence, patient characteristics and survival over 18 years in Perth. Western Australia Resuscitation Plus.

[3] Daya M.R., Schmicker R.H., Zive D.M. (2015). Out-of-hospital cardiac arrest survival improving over time: Results from the Resuscitation Outcomes Consortium (ROC). Resuscitation.

[4] Nehme Z., Andrew E., Bernard S., Smith K. (2015). Comparison of out-of-hospital cardiac arrest occurring before and after paramedic arrival: Epidemiology, survival to hospital discharge and 12-month functional recovery. Resuscitation.

[5] Chia M.Y.C., Kwa T.P.W., Wah W. (2019). Comparison of outcomes and characteristics of emergency medical services (EMS)-witnessed, bystander-witnessed, and unwitnessed out-of-hospital cardiac arrests in Singapore. Prehospital Emergency Care.

[6] Hostler D., Thomas E.G., Emerson S.S. (2010). Increased survival after EMS witnessed cardiac arrest. Observations from the Resuscitation Outcomes Consortium (ROC) Epistry—Cardiac arrest. Resuscitation.

[7] Sasson C., Rogers M.A.M., Dahl J., Kellermann A.L. (2010). Predictors of survival from out-of-hospital cardiac arrest. Circulation: Cardiovascular Quality and Outcomes.

[8] Perkins G.D., Jacobs I.G., Nadkarni V.M. (2015). Cardiac arrest and cardiopulmonary resuscitation outcome reports: update of the utstein resuscitation registry templates for out-of-hospital cardiac arrest. Circulation.

[9] Geoscience Australia. Area of Australia - States and Territories [cited 23 Mar 2023]. Available from: https://www.ga.gov.au/scientific-topics/national-location-information/dimensions/area-of-australia-states-and-territories.

[10] Australian Bureau of Statistics (ABS). National, state and territory population: Statistics about the population and components of change (births, deaths, migration) for Australia and its states and territories 2023 [cited 13 Mar 2023]. Available from: https://www.abs.gov.au/statistics/people/population/national-state-and-territory-population/latest-release.

[11] Australian Bureau of Statistics (ABS). Greater Perth: 2016 Census All persons QuickStats [cited 23 Mar 2023]. Available from: https://www.abs.gov.au/census/find-census-data/quickstats/2016/5GPER.

[12] St John WA. Annual Report FY22/23 [cited 9 Jan 2024]. Available from: https://news.stjohnwa.com.au/wp-content/uploads/2023/10/FY22-23-Annual-Report.pdf.

[13] Talikowska M., Ball S., Whiteside A., Belcher J., Finn J. (2023). Use of dispatch codes for obvious/expected deaths: Maintaining patient safety while reducing the number of lights-and-sirens responses. Resuscitation.

[14] St John WA. Clinical Resources: Determination of Death (TOR/ROLE) [updated August 2021;cited 6th July 2023]. Available from: https://clinical.stjohnwa.com.au/clinical-practice-guidelines/circulation/determination-of-death-(tor-role).

[15] St John WA. Out-of-Hospital Cardiac Arrest Report 2020 [cited 23 Mar 2023]. Available from: https://impact.stjohnwa.com.au/docs/default-source/default-document-library/ohca-report-2020_web.pdf?sfvrsn=9a6f6652_0.

[16] Song J., Guo W., Lu X., Kang X., Song Y., Gong D. (2018). The effect of bystander cardiopulmonary resuscitation on the survival of out-of-hospital cardiac arrests: a systematic review and meta-analysis. Scandinavian Journal of Trauma, Resuscitation and Emergency Medicine.

[17] Priority Dispatch (2019). Medical Priority Dispatch System (MPDS) Information Sheet. [cited 4th May 2020]. Available from: https://prioritydispatch-media.s3.amazonaws.com/prioritydispatch.net/salessheets/PDC_MPDS_Sales_Sheet_v8_web.pdf.

[18] Dunn O.J. (1961). Multiple Comparisons among Means. Journal of the American Statistical Association.

[19] Ranganathan P., Pramesh C.S., Aggarwal R. (2017). Common pitfalls in statistical analysis: Logistic regression. Perspect Clin Res.

[20] Al-Dury N., Ravn-Fischer A., Hollenberg J. (2020). Identifying the relative importance of predictors of survival in out of hospital cardiac arrest: a machine learning study. Scandinavian Journal of Trauma, Resuscitation and Emergency Medicine.

[21] Hessulf F., Bhatt D.L., Engdahl J. (2023). Predicting survival and neurological outcome in out-of-hospital cardiac arrest using machine learning: the SCARS model. eBioMedicine.

[22] Andersen L.W., Grossestreuer A.V., Donnino M.W. (2018). “Resuscitation time bias”—A unique challenge for observational cardiac arrest research. Resuscitation.

[23] Gold L.S., Eisenberg M.S. (2010). A comprehensive investigation of cardiac arrest before and after arrival of emergency medical services. Resuscitation.

[24] De Maio V.J., Stiell I.G., Wells G.A., Spaite D.W. (2000). Cardiac arrest witnessed by emergency medical services personnel: descriptive epidemiology, prodromal symptoms, and predictors of survival. Annals of Emergency Medicine.

[25] Nehme Z., Andrew E., Bray J.E. (2015). The significance of pre-arrest factors in out-of-hospital cardiac arrests witnessed by emergency medical services: A report from the Victorian Ambulance Cardiac Arrest Registry. Resuscitation.

[26] Skogvoll E., Eftestøl T., Gundersen K. (2008). Dynamics and state transitions during resuscitation in out-of-hospital cardiac arrest. Resuscitation.

[27] Kvaløy J.T., Skogvoll E., Eftestøl T. (2009). Which factors influence spontaneous state transitions during resuscitation?. Resuscitation.

[28] Holmström L., Reinier K., Toft L. (2022). Out-of-hospital cardiac arrest with onset witnessed by emergency medical services: Implications for improvement in overall survival. Resuscitation.

[29] Bergström M., Schmidbauer S., Herlitz J., Rawshani A., Friberg H. (2018). Pulseless electrical activity is associated with improved survival in out-of-hospital cardiac arrest with initial non-shockable rhythm. Resuscitation.

[30] Majewski D., Ball S., Finn J. (2019). Systematic review of the relationship between comorbidity and out-of-hospital cardiac arrest outcomes. BMJ Open.

