# Supplementary material for: Do differences in emergency medical services (EMS) response time to an arrest account for the survival differences between EMS-witnessed and bystander-witnessed out of hospital cardiac arrest?
Source: Resusc Plus. 2024 Jun 25;19:100696. doi: 10.1016/j.resplu.2024.100696 (PMC11259960; doi:10.1016/j.resplu.2024.100696)
Supplement: Supplementary Data 1 [file mmc1.docx]

|  |  | **Arrest witness status** | | | | |
| --- | --- | --- | --- | --- | --- | --- |
|  |  | **EMS-witnessed**  **(n = 510)** | **Bystander-witnessed**  **No bystander CPR**  **(n = 302)** | **p-value^a^** | **Bystander-witnessed**  **with bystander CPR**  **(n = 1318)** | **p-value^b^** |
| **Advanced airway^a^** | **Yes** | 259 (50.8 %) | 238 (78.8%) | P<0.001* | 948 (71.9%) | P<0.001* |
|  | **No** | 251 (49.2%) | 64 (21.2%) |  | 370 (28.1%) |  |
| **Cardiac arrest drugs administered^b^** | **Yes** | 255 (50.0%) | 245 (81.1%) | P<0.001* | 947 (71.9%) | P<0.001* |
|  | **No** | 255 (50.0%) | 57 (18.9%) |  | 371 (28.1%) |  |
