# Supplementary material for: Do differences in emergency medical services (EMS) response time to an arrest account for the survival differences between EMS-witnessed and bystander-witnessed out of hospital cardiac arrest?
Source: Resusc Plus. 2024 Jun 25;19:100696. doi: 10.1016/j.resplu.2024.100696 (PMC11259960; doi:10.1016/j.resplu.2024.100696)
Supplement: Supplementary Data 2 [file mmc2.docx]

**Sensitivity analysis:**

|  | **EMS-witnessed**  Unknown shockable: 8  Unknown non-shockable: 21 | | | **Bystander-witnessed (with bystander CPR)**  Unknown shockable: 182  Unknown non-shockable: 57 | | |
| --- | --- | --- | --- | --- | --- | --- |
|  | Reported | Potential Max | Potential Min | Reported | Potential Max | Potential Min |
| PEA, n (%) | 210 (44.8%) | 231 (46.4%) | 210 (42.2%) | 174 (15.6%) | 231 (17.5%) | 174 (13.2%) |
| Asystole, n (%) | 108 (23.0%) | 129 (25.9%) | 108 (21.7%) | 453 (34.4%) | 510 (38.7%) | 453 (34.4%) |

Min EMS-witnessed PEA, and Max bystander-witnessed PEA: 42.2% vs 17.5%, p<0.001*

* Chi-squared test
